# ART history prior to conception: trends and association with postpartum disengagement from HIV care in Khayelitsha, South Africa (2013–2019): a retrospective cohort study

**DOI:** 10.1002/jia2.26236

**Published:** 2024-04-02

**Authors:** Tamsin Kate Phillips, Reshma Kassanjee, Nicola Maxwell, Kim Anderson, Leigh Johnson, Haroon Moolla, Landon Myer, Benjamin H. Chi, Jonathan Euvrard, Andrew Boulle, Mary‐Ann Davies, Morna Cornell, Renee de Waal

**Affiliations:** ^1^ Centre for Infectious Disease Epidemiology & Research School of Public Health University of Cape Town Cape Town South Africa; ^2^ Division of Epidemiology & Biostatistics School of Public Health University of Cape Town Cape Town South Africa; ^3^ Department of Obstetrics and Gynecology School of Medicine University of North Carolina at Chapel Hill Chapel Hill North Carolina USA; ^4^ Department of Health and Wellness Provincial Government of the Western Cape Cape Town South Africa

**Keywords:** disengagement from care, return to care, retention, antiretroviral therapy, postpartum, South Africa

## Abstract

**Introduction:**

In recent years, the expansion of HIV treatment eligibility has resulted in an increase in people with antiretroviral therapy (ART) experience prior to pregnancy but little is known about postpartum engagement in care in this population. We examined differences in disengagement from HIV care after delivery by maternal ART history before conception.

**Methods:**

We analysed data from people living with HIV (aged 15–49) in Khayelitsha, South Africa, with ≥1 live birth between April 2013 and March 2019. We described trends over time in ART history prior to estimated conception, classifying ART history groups as: (A) *on ART* with no disengagement (>270 days with no evidence of HIV care); (B) *returned* before pregnancy following disengagement; (C) *restarted* ART in pregnancy after disengagement; and (D) ART *new start* in pregnancy. We used Kaplan–Meier curves and proportional‐hazards models (adjusted for maternal age, number of pregnancy records and year of delivery) to examine the time to disengagement from delivery to 2 years postpartum.

**Results:**

Among 7309 pregnancies (in 6680 individuals), the proportion *on ART* (A) increased from 19% in 2013 to 41% in 2019. The proportions of those who *returned* (B) and *restarted* (C) increased from 2% to 13% and from 2% to 10%, respectively. There was a corresponding decline in the proportion of *new starts* (D) from 77% in 2013 to 36% in 2019. In the first recorded pregnancy per person in the study period, 26% (95% CI 25–27%) had disengaged from care by 1 year and 34% (95% CI 33–36%) by 2 years postpartum. Individuals who *returned* (B: aHR 2.10, 95% CI 1.70–2.60), *restarted* (C: aHR 3.32, 95% CI 2.70–4.09) and *newly started ART* (D: aHR 2.41, 95% CI 2.12–2.74) had increased hazards of postpartum disengagement compared to those *on ART* (A).

**Conclusions:**

There is a growing population of people with ART experience prior to conception and postpartum disengagement varies substantially by ART history. Antenatal care presents an important opportunity to understand prior ART experiences and an entry into interventions for strengthened engagement in HIV care.

## 1 INTRODUCTION

Although antiretroviral therapy (ART) is recommended for all people living with HIV (PLHIV), major challenges persist with engagement in care [1, 2]. For pregnant PLHIV, the benefits of lifelong ART include maintaining their own health, preventing sexual transmission to partners and preventing vertical transmission of HIV to infants [3]. However, these individual‐ and population‐level benefits are threatened by disengagement from HIV care [4, 5, 6].

Postpartum disengagement is well‐documented among people who start ART during pregnancy [4]. Pregnancy has historically been a common entry point into HIV care and vertical transmission prevention programmes across sub‐Saharan Africa have been very successful at identifying and initiating ART among pregnant PLHIV [7]. This, together with the rapid implementation of universal ART, means that over time an increasing proportion of people have conceived subsequent to ART initiation [8]. Studies before the introduction of universal ART found no difference in postpartum disengagement among those starting ART in pregnancy versus those conceiving on ART [9, 10], but there are few data on these outcomes in more recent years.

In this study, we characterized trends in the timing of ART initiation and engagement history before conception in a large HIV cohort in Khayelitsha, South Africa. We also examined differences in postpartum disengagement according to ART history. As we work towards the global goals of eliminating vertical HIV transmission and ending AIDS by 2030 [11], understanding outcomes in ART‐experienced pregnant PLHIV will be critical to closing the remaining gaps.

## 2 METHODS

### 2.1 Setting

Khayelitsha, in Cape Town, has an estimated population of > 500,000 people and almost 40% unemployment. The area has a high HIV prevalence (antenatal prevalence 31% in 2017 [12]) and a large public‐sector ART programme provides free HIV care to ∼50,000 people [13].

Eligibility criteria to initiate lifelong ART in the Western Cape province have changed over time in line with national and global guidelines. In Khayelitsha, ART became available in 2001, with strict eligibility criteria [13]. From 2011, adults with CD4 cell counts <350 cells/µl or WHO Stage III or IV disease were eligible to start ART [14]. In April 2013, Western Cape guidelines changed to allow all people with tuberculosis and all pregnant and breastfeeding people to start lifelong ART regardless of CD4 cell count or clinical stage [15]. In April 2015, the CD4 cell count threshold for non‐pregnant adults shifted to 500 cells/µl, and in September 2016, universal ART was introduced [13]. Fixed dose combination tenofovir, emtricitabine/lamivudine and efavirenz was recommended as the preferred first‐line ART regimen between 2013 and 2019 [16], replaced by fixed dose tenofovir, lamivudine and dolutegravir in December 2019 [17].

Antenatal care is offered in the same primary care clinics as HIV treatment until the third trimester, when care is transferred to dedicated Midwife Obstetric Units. Opt‐out HIV testing is offered to all pregnant people and integrated HIV and antenatal care is provided. Those with complications or requiring caesarean section are referred to secondary or tertiary hospitals for delivery. Following delivery, mothers are referred to primary care clinics to continue their HIV care. Mothers and infants may attend care at the same or separate primary care clinics postpartum.

### 2.2 Data sources

This study used data from the Khayelitsha cohort of the International epidemiology Databases to Evaluate AIDS Southern Africa (IeDEA‐SA) collaboration [18]. PLHIV enter the cohort when they access HIV services at public sector ART sites in Khayelitsha, which contribute to an anonymized dataset. IeDEA‐SA data included treatment history, CD4 cell count and HIV viral load test dates and results. In the Western Cape, public‐sector patient data are collated across health facilities by the Western Cape Provincial Health Data Centre (PHDC) using unique patient identifiers [19]. We obtained additional ART dispensing and pregnancy data from the PHDC for those in the cohort. Deaths were ascertained through facility‐recorded deaths and by linking to the South African National Population Register (NPR) which provides vital status details for people with recorded national identification numbers [20, 21]. Data on gestational age at delivery were not available and the conception date was estimated as the delivery date minus 40 weeks.

### 2.3 Disengagement definition

Disengagement from care was defined as having a period of >270 days with no evidence of HIV care (no ART dispensing, CD4 cell count or HIV viral load test) at any public‐sector Western Cape facility (based on linked PHDC data). PLHIV in Khayelitsha routinely receive 2–4 months’ ART supply at each visit, with some differentiated service delivery models supplying up to 6 months’ ART. The quantity of ART dispensed is not always reflected in the routine data. Thus, a period of >270 days (9 months) with no evidence of care was chosen as a conservative definition, robust to discrepancies in dispensing quantities.

### 2.4 Comparison groups

Pregnant PLHIV were categorized into one of four groups based on ART history in relation to pregnancy (Table 1). PLHIV were considered ART‐experienced if their ART initiation date was >270 days before estimated conception. This window was chosen to allow people to be established on ART and to have had time to potentially disengage from care before the pregnancy. Those who started ART for the first time ≤270 days before estimated conception were excluded from primary analyses (*n* = 630) but considered in a sensitivity analysis. ART‐experienced PLHIV were further divided based on history of disengagement: on ART at conception with no previous disengagement (*A. On ART*), disengaged at least once and returned to care before conception (*B. Returned*) and restarting ART during pregnancy after disengaging from care before conception (*C. Restarted*). New ART initiations were defined as PLHIV who had no history of ART use before conception and who started ART between the estimated conception date and up to 2 weeks after delivery to capture any delayed initiations (*D. New start)*.

**Table 1:** Definitions of antiretroviral therapy (ART) history prior to estimated conception^a^

ART history group		Description	Definition
**Has ART experience before the estimated conception date**	**A. On ART**	Has ART history, engaged in care from ART initiation to conception	ART started >270 days prior to the estimated conception date with no evidence of any period of >270 days with no HIV care between ART initiation and estimated conception
	**B. Returned**	Has ART history, disengaged at least once and returned to care before conception	ART started >270 days prior to the estimated conception date, has evidence of at least one period of >270 days with no HIV care, and has evidence of accessing HIV care in the 270 days prior to estimated conception and during pregnancy
	**C. Restarted**	Has ART history, disengaged at least once and is not in care at conception	ART started >270 days prior to the estimated conception date, has evidence of at least one period of >270 days with no HIV care, has no evidence of accessing HIV care in the 270 days prior to estimated conception but has evidence of restarting ART between estimated conception and 2 weeks after delivery
**Has no ART experience before the estimated conception date**	**D. New start**	Started ART during the pregnancy for the first time	First evidence of ART initiation between estimated conception and 2 weeks after delivery

^a^ Estimated conception was calculated as delivery date minus 40 weeks.

### 2.5 Eligibility criteria

We included PLHIV who had a record of initiating ART and having ≥1 live birth between 1 April 2013 and 31 March 2019. Pregnancies were excluded if the person was <15 years old at the estimated conception date (*n* = 10), if the ART start date was >2 weeks after delivery (i.e. outside the perinatal period, *n* = 2078), if there was no evidence of any HIV care during pregnancy (*n* = 74) or if the person was transferred out of the cohort before delivery (*n* = 288). For sensitivity analyses including repeat pregnancies (>1 pregnancy in the study period), subsequent pregnancies were included if there were ≥10 months between delivery dates.

### 2.6 Key variables

The primary outcome was time to first disengagement from care after delivery, based on our *a priori* definition, up to 2 years postpartum. At this point, individuals were censored regardless of whether they later returned.

Other covariates included in analyses were age at delivery, first evidence of a pregnancy (first or repeat pregnancy based on all pregnancies digitized in the PHDC, not restricted to the study period) and year of delivery. CD4 cell count nearest to first ART initiation (within 6 months before to 2 weeks after ART initiation) and, for those ART‐experienced, viral load in the year before estimated conception and duration on ART at delivery were also described.

### 2.7 Statistical analysis

Analyses were conducted in Stata v17.0. We described cohort characteristics using frequencies and proportions or medians with interquartile ranges (IQR) as appropriate. We described ART history before estimated conception by year of delivery, including all pregnancies. Primary time‐to‐event analyses included the first delivery recorded in the study period (April 2013–March 2019) and repeat pregnancies were included in sensitivity analyses. Individuals entered analyses at delivery date and, per pregnancy, exited analyses at the earlier of 2 years postpartum or 270 days before 31 March 2020 (database closure). This was done to ensure that 270 days were observed before database closure and all individuals had the potential to disengage from care postpartum [22]. Per pregnancy, individuals were censored at the earliest of transfer out, death or analysis closure. Due to very small numbers of deaths, deaths were censored and not treated as a competing event. Disengagement from care (no evidence of HIV care for >270 days regardless of evidence of care after this period) was the failure event with failure assigned to the date of last evidence of HIV care before disengagement. If disengagement occurred directly after delivery, the failure date was assigned to the delivery date plus 1 day [22].

Kaplan–Meier curves were used to examine the cumulative probability of disengaging from care after delivery, stratified by ART history group. Predictors of time to first disengagement were assessed using crude and adjusted Cox proportional‐hazards models, with ART history as the primary exposure. Other covariates included in adjusted models were maternal age at delivery, whether it was the first or repeat pregnancy ever recorded in the PHDC and year of delivery. The proportional hazards assumption was met for all variables except the year of delivery, which was included as a stratifying variable, allowing the baseline hazard to differ by year of delivery in adjusted models [23]. In a sub‐analysis of ART‐experienced groups (excluding group D, *new starts*), we also adjusted for the time between the first ART initiation and delivery.

We conducted sensitivity analyses to explore changes in the model results when: (i) including repeat pregnancies during the study period; (ii) using a shorter disengagement definition of >180 days with no evidence of HIV care (the maximum period of ART dispensed in this setting); (iii) including available CD4 cell count at first ART initiation; (iv) excluding people with less than 2 years postpartum follow up; and (v) including the group whose first recorded ART initiation date was ≤270 days before estimated conception. In Cox proportional‐hazards models including repeat pregnancies, observation time for each pregnancy was censored at the next estimated conception date (if prior to 2 years postpartum) and cluster‐robust standard errors were calculated to allow for intra‐individual correlation [23].

### 2.8 Ethics

IeDEA Southern Africa and the Khayelitsha cohort have approval from the Human Research Ethics Committee of the University of Cape Town to contribute routine, anonymized, individual‐level data for research within the IeDEA collaboration. These data were not identifiable and no individual informed consent was obtained.

## 3 RESULTS

**Figure 1:**
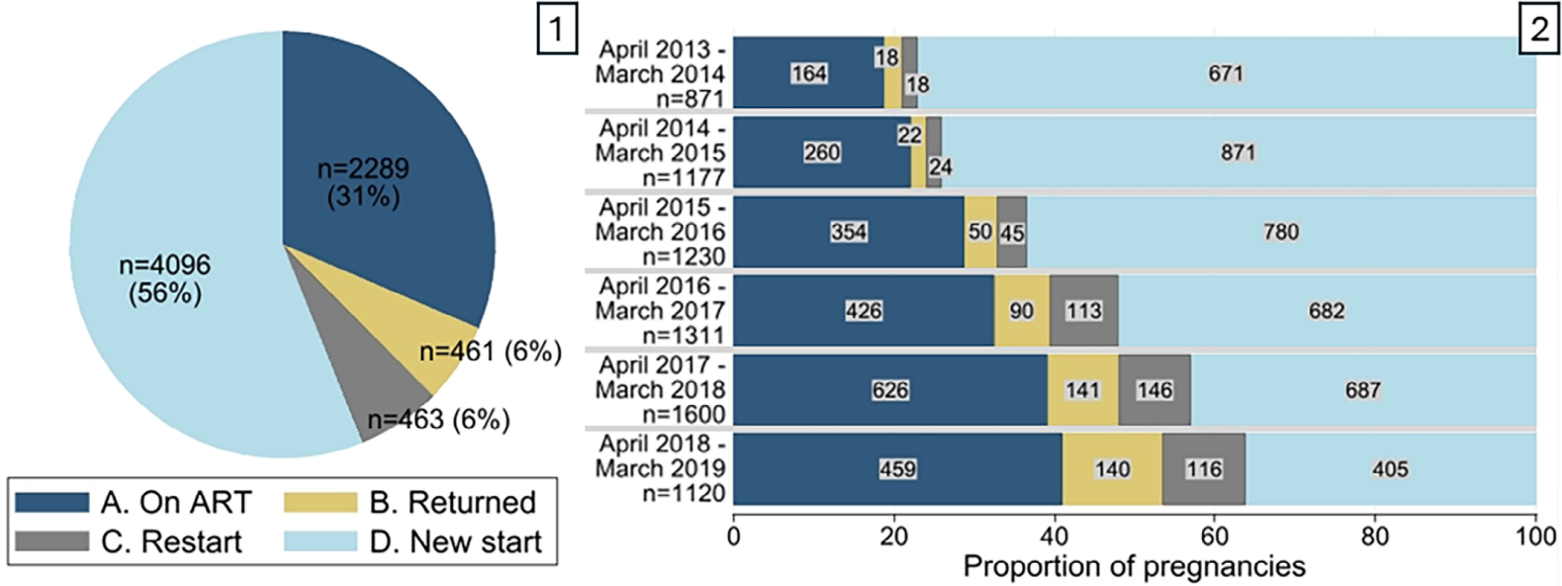
The overall distribution of antiretroviral therapy (ART) history groups (1) and the trend in the proportion of pregnancies in each ART history group by year of delivery (2) for 7309 pregnancies in 6680 people living with HIV from April 2013 through March 2019.

There were 9481 PLHIV who had a recorded ART initiation date and at least one pregnancy in the study period, with 10,389 pregnancies recorded in total. Of these, 3080 pregnancies were excluded (described above) and 7309 pregnancies from 6680 PLHIV were included. Median age at delivery was 28.4 years and for 74%, there was no digital record of an earlier pregnancy (Table 2). Overall, 31% (*n* = 2289) of all pregnancies occurred in PLHIV *on ART* (A), 6% (*n* = 461) had *returned* (B), 6% (*n* = 463) *restarted* ART (C) and 56% (4096) were *new starts* (D) (Table 2 and Figure 1). Viral loads in the year prior to estimated conception were available in 83% and 66% of pregnancies among those *on ART* and *returned*, respectively; 96% and 82% of these viral loads were <400 copies/ml.

**Table 2:** Characteristics of 6680 persons living with HIV with 7309 pregnancies between April 2013 and March 2019, grouped by antiretroviral therapy (ART) history prior to conception: (A) On ART, (B) Returned, (C) Restarting ART and (D) New ART start

	Total	(A) On ART	(B) Returned	(C) Restarted	(D) New start
**First pregnancy in the period April 2013–March 2019**	**6680**	**1912 (29)**	**381 (6)**	**291 (4)**	**4096 (61)**
Median maternal age at first ART initiation (IQR)	28.3 (24.7–32.5)	30.4 (26.6–34.1)	29.6 (26.7–33.5)	28.7 (25.3–32.4)	27.1 (23.9–31.3)
Pregnancies in the period April 2013–March 2019					
More than one	605 (9)	120 (6)	17 (4)	21 (7)	447 (11)
Only one	6075 (91)	1792 (94)	364 (96)	270 (93)	3649 (89)
First recorded pregnancy^a^					
First pregnancy	5391 (81)	1475 (77)	288 (76)	199 (68)	3429 (84)
Not first pregnancy	1289 (19)	437 (23)	93 (24)	92 (32)	667 (16)
Median years from first ART start to delivery (IQR)	0.6 (0.4–3.0)	3.0 (2.2–4.3)	4.6 (3.5–5.8)	3.6 (2.7–4.8)	0.4 (0.2–0.5)
Number with a CD4 count near first ART start^b^	6016 (90)	1751 (92)	309 (81)	263 (90)	3693 (90)
Median CD4 cells/µl (IQR)	325 (217–475)	266 (172–345)	234 (144–330)	282 (199–367)	382 (255–535)
CD4 categories					
<200	1291 (21)	562 (32)	126 (41)	66 (25)	537 (15)
≥200, <350	2066 (34)	769 (44)	116 (38)	120 (46)	1061 (29)
≥350, <500	1347 (22)	299 (17)	30 (10)	41 (16)	977 (26)
≥500	1312 (22)	121 (7)	37 (12)	36 (14)	1118 (30)
Number linked to National Population Register	4894 (73)	1238 (65)	276 (72)	212 (73)	3168 (77)
Death recorded during the analysis period^c^	40 (0.6)	8 (0.4)	3 (0.8)	4 (1.4)	25 (0.6)
**All pregnancies from April 2013 to March 2019**	**7309**	**2289 (31)**	**461 (6)**	**463 (6)**	**4096 (56)**
Median maternal age at delivery (IQR)	28.4 (24.8–32.5)	30.3 (26.6–34.1)	29.8 (26.8–33.4)	28.3 (25.1–31.9)	27.1 (23.9–31.3)
First recorded pregnancy^a^					
First pregnancy	5391 (74)	1475 (64)	288 (62)	199 (43)	3429 (84)
Not first pregnancy	1918 (26)	814 (36)	173 (38)	264 (57)	667 (16)
Median years from first ART start to delivery (IQR)	0.5 (0.3–2.7)	3.0 (2.2–4.2)	4.6 (3.4–5.8)	3.8 (2.8–5.3)	0.4 (0.2–0.5)
Number with viral load in year prior to conception^d^	–	1891 (83)	303 (66)	30 (6)	0 (0)
Viral load categories					
<400	–	1806 (96)	248 (82)	23 (77)	–
≥400, <1000	–	23 (1)	9 (3)	3 (10)	–
≥1000	–	62 (3)	46 (15)	4 (13)	–

*Note*: Results presented as *n* (%) or median and interquartile range where specified.

^a^ First digital evidence of pregnancy based on all pregnancies recorded in the Provincial Health Data Centre.

^b^ CD4 cell count taken within 6 months prior and up to 2 weeks after the ART initiation date.

^c^ Deaths include facility recorded deaths from the routine data and additional death identified in the National Population Register. There were 40 deaths in between delivery and database closure. Two people were transferred out of the cohort prior to death and 10 people disengaged from care prior to death, so only 28 had death as an outcome in the time‐to‐event analyses.

^d^ All viral loads in group C (restarted) were taken >270 days prior to the estimated conception date.

Based on the first pregnancy per person in the study period (*n* = 6680, 81% no evidence of an earlier pregnancy), the median age at ART initiation was 26.6 years (IQR 23.3–30.5) and the *returned* and *restarted* groups were younger compared to people *on ART* and *new starts* (Table 2). Median CD4 cell count nearest ART initiation was 325 cells/µl (IQR 217–475; CD4 cell counts were available for 90% of the cohort with no substantial differences between those with and without a CD4 cell count, Table S1). CD4 cell count at ART initiation was higher among *new starts* compared to the three ART‐experienced groups. There were 40 maternal deaths between delivery and database closure (70% of individuals could be linked to the NPR, Table S1). Duration between first ART initiation and delivery was greatest for pregnancies among those who *returned* to care before conception (median 4.6 years IQR 3.5–5.8), followed by those who *restarted* in pregnancy (median 3.6 years IQR 2.7–4.8) and those *on ART* (median 3.0 years IQR 2.2–4.2). Median duration on ART at delivery was 0.4 years (IQR 0.2–0.5) among *new starts* in pregnancy.

### 3.1 Trends in ART history prior to pregnancy over time

From 2013 to 2019, there was a steady decline in the proportion of pregnancies in which PLHIV newly initiated ART (D: 77% to 36%, Figure 1), and an increase in the proportion of pregnancies among people *on ART* (A: 19–41%), those who *returned* to care before conception (B: 2–13%) and ART *restarts* after conception (C: 2–10%). Among pregnancies in those who *returned* to care prior to conception, 16% (*n* = 76) had evidence of two or more periods of disengagement prior to the incident pregnancy. Of the pregnancies among *restarts* (*n* = 463), 22% (*n* = 100) also had an earlier disengagement with return to care.

### 3.2 Time to first postpartum disengagement from care

**Figure 2:**
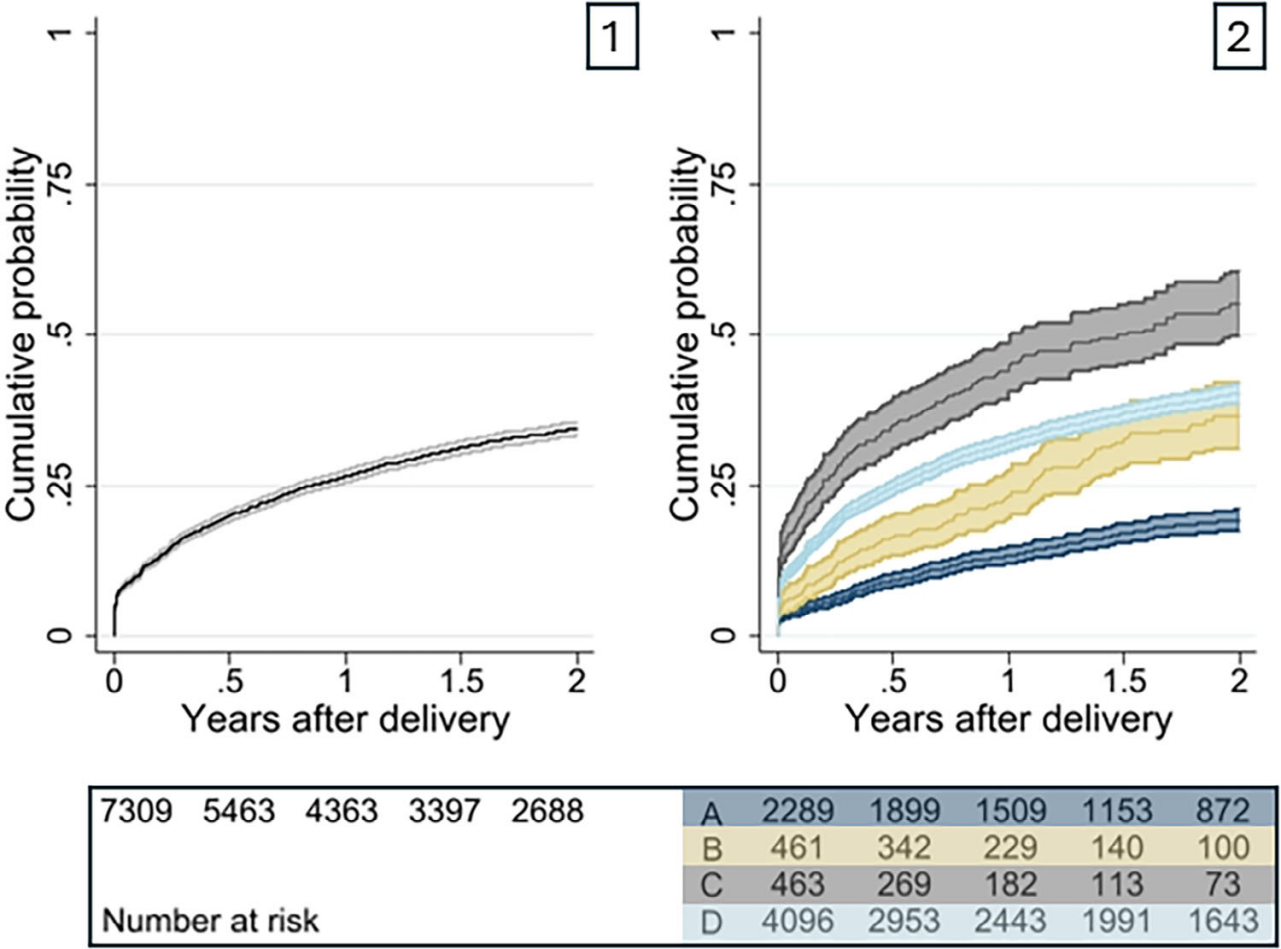
Kaplan–Meier failure curves showing the cumulative probability of individuals disengaging from HIV care after delivery (>270 days with no evidence of HIV care), overall (1) and by antiretroviral therapy (ART) history group prior to conception (2): (A) On ART, (B) Returned, (C) Restart and (D) New start.

At 1 and 2 years postpartum, 26% (95% CI 25–27%) and 34% (95% CI 33–36%) of people had disengaged from care, respectively. The probability of disengagement differed by ART history (Figure 2). Disengagement was highest in the *restarted* group (C: 40%, 95% CI 34–46%), followed by *new starts* (D: 32%, 95% CI 31–34%) and those who had *returned* (B: 23%, 95% CI 19–28%). Disengagement was lowest among those *on ART* (A: 12%, 95% CI 11–14%). By 2 years postpartum, the probability of disengaging from care was 51% (95% CI 44–58%) among *restarts*, 40% (95% CI 39–42%) among *new starts*, 36% (31–43%) among those who had *returned* and 18% (95% CI 16–20%) among those *on ART*.

These differences persisted in multivariable models (Table 3). Compared to people *on ART* (A), *restarts* during pregnancy (C: aHR 3.32, 95% CI 2.70–4.09), those who had disengaged but *returned* to care pre‐conception (B: aHR 2.10, 95% CI 1.70–2.60) and *new starts* in pregnancy (D: aHR 2.41, 95% CI 2.12–2.74) all had increased hazards of disengaging from care postpartum.

**Table 3:** Estimated hazard ratios from Cox‐proportional hazards models predicting time to first disengagement from care after delivery (a period of >270 days with no evidence of HIV care), for the first pregnancy in the period April 2013–March 2019 (*n* = 6680)

	Crude HR (CI)	Adjusted^a^ HR (CI)
ART history prior to conception		
A. On ART	Ref	Ref
B. Returned	2.10 (1.68–2.62)	2.10 (1.70–2.60)
C. Restart	3.61 (2.93–4.45)	3.32 (2.70–4.09)
D. New start	2.72 (2.40–3.08)	2.41 (2.12–2.74)
Age at delivery (per 5 additional years of age)	0.75 (0.72–0.77)	0.80 (0.76–0.84)
First recorded pregnancy^b^		
Not first pregnancy	Ref	Ref
First pregnancy	0.91 (0.82–1.02)	0.82 (0.73–0.91)
Year of delivery		
April 2013–March 2014	Ref	
April 2014–March 2015	0.99 (0.84–1.17)	
April 2015–March 2016	1.11 (0.95–1.31)	
April 2016–March 2017	1.00 (0.85–1.17)	
April 2017–March 2018	1.03 (0.87–1.21)	
April 2018–March 2019	0.63 (0.52–0.76)	

Abbreviations: aHR, adjusted hazard ratio; ART, antiretroviral therapy; CI, 95% confidence interval.

^a^ In the adjusted model, year of delivery is not shown as it was treated as a stratifying variable allowing the baseline hazard to vary by year.

^b^ First digital evidence of pregnancy based on all pregnancies recorded in the Provincial Health Data Centre.

In sensitivity analyses (Table S2), the findings did not change substantially when including eligible repeat pregnancies in the study period, when disengagement was defined as >180 days with no evidence of HIV care, or when available CD4 cell count at first ART initiation was included in the model. Estimated hazard ratios for postpartum disengagement were slightly higher in a sub‐analysis of ART‐experienced individuals, adjusting for years between ART initiation and delivery (Table S3), but the 95% CI overlapped with those in the adjusted model including all pregnant PLHIV (Table 3): aHR 2.49 (95% CI 1.98–3.14) for those who *returned* prior to pregnancy and 3.84 (95% CI 3.06–4.81) among *restarts* in pregnancy, compared to those *on ART*. Excluding people with less than 2 years of postpartum observation (Table S4) did not substantially alter the association between ART history and postpartum engagement. Results were also consistent when the group with recent ART initiation (≤270 days prior to estimated conception) was included and this group had a two‐fold higher hazard of disengaging postpartum (aHR 2.01 95% CI 1.68–2.39) compared to pregnant PLHIV *on ART* (Table S5).

## 4 DISCUSSION

Our findings highlight that many PLHIV are now ART‐experienced at the time of conception, following the scale‐up of lifelong ART over the past decade. Reassuringly, in this analysis, most ART‐experienced pregnant PLHIV appeared to have remained on ART without periods of disengagement. There were, however, two small but growing groups who had disengaged from care prior to conception: those who had returned to care before conception, and those restarting ART in pregnancy. Both groups, and particularly those restarting ART, had a substantially increased hazard of postpartum disengagement compared to those on ART without a history of disengagement. While the proportion of people newly starting ART in pregnancy is declining, this group was also at increased risk of disengaging postpartum compared to those on ART with no disengagement.

ART non‐adherence while breastfeeding remains a substantial contributor to new paediatric HIV acquisitions thus this period is critical for the prevention of vertical HIV transmission [24, 25]. Postpartum disengagement was high in all groups in our study, even using a conservative window of >270 days without HIV care. Half of people restarting ART in pregnancy and a third of people returning before conception had disengaged by 2 years postpartum, adding to the growing evidence that people who return after an interruption remain at risk for future interruptions and poor outcomes [26, 27, 28]. In line with previous research on postpartum retention among pregnant PLHIV starting ART since the rollout of Option B+ [4], 40% of pregnant PLHIV newly starting ART had disengaged by 2 years postpartum. While the incidence of disengagement was lowest among people on ART at conception without disengagement, it is concerning that 18% of this group had disengaged by 2 years. Consistent with the existing literature, younger age was associated with an increased hazard of postpartum disengagement [4, 29, 30].

There is clearly a broad need for cost‐effective and sustainable interventions to support engagement in care postpartum, and more intense interventions may be needed to support young mothers, those newly starting ART, and those with a history of disengagement. Systematic reviews of interventions to improve engagement in HIV care among women [31], among pregnant and postpartum PLHIV [32, 33], and among young pregnant and postpartum PLHIV specifically [34] have shown mixed results on effective strategies. Promising approaches include community and family‐centred interventions, peer‐led and mobile phone‐based support, and enhanced counselling [31, 33]. Integration of maternal and child HIV and postnatal care is recommended and has been shown to improve postpartum outcomes [35, 36, 37], but implementation remains varied [38]. Differentiated models of care require consideration for pregnant and breastfeeding PLHIV and people at risk of disengagement [39, 40]. Community adherence clubs have shown improved viral load outcomes at 24 months postpartum compared to standard clinic care for people whose viral loads were suppressed at delivery [41]. Integrated postnatal clubs for postpartum PLHIV have also been implemented in South Africa with improved infant testing uptake and maternal retention compared to historical controls [42]. Postnatal clubs appear to be feasible and acceptable to patients and providers [43, 44] and the mix of patient age and treatment experience in these groups may provide a valuable opportunity for peer and social support. Postpartum interventions could be combined in multicomponent, stepped or menu‐based approaches, and future research should carefully consider implementation context and patient preferences [45, 46, 47].

Our study provides recent data on differences in outcomes between people newly starting ART in pregnancy and those with ART‐experience before conception. An early study from Switzerland (1996–2011) did not find a statistically significant association between being on ART at conception and the odds of postpartum loss to follow‐up (defined as no visit for 1 year after delivery; adjusted odds ratio for loss to follow‐up among those on ART at conception vs. not on ART 0.63, 95% CI 0.34–1.14) [9]. A South African study before the rollout of Option B+ and universal ART (2004–2013) did not observe associations with loss to follow‐up (more than 3 months late for a visit; aHR 0.9, 95% CI 0.7–1.1) but found a higher hazard of postpartum virologic failure among those already on ART versus those starting ART in pregnancy (aHR 1.8 95% CI 1.1–2.7) [10]. Substantial changes in available ART regimens, ART eligibility and models of care in more recent years make it difficult to directly compare these findings.

An important strength of our study is the robust routine data maintained by the Western Cape PHDC that links all public sector facilities across the province—and multiple health information systems—using unique patient identifiers [19]. In this setting where people are known to move between clinics postpartum [48], the PHDC data allowed ascertainment of HIV care access regardless of where care was received across a wide geographic area. This is a common challenge in studies of facility‐specific engagement in care, particularly where transfer of clinic is required [49]. We also note some limitations. Mobility may have resulted in an overestimate of disengagement. If individuals accessed HIV care outside of the Western Cape or in the private sector, this would not have been detected. In prior analyses of postpartum mobility in Gugulethu, an area near Khayelitsha with a similar population demographic, 9% of people had moved out of the Western Cape in the first 2 years after pregnancy [48]. However, while some people do connect to care in other provinces, mobility is also a risk factor for disengagement [48, 50] and even with some misclassification, disengagement in this cohort remains high. Similarly, some ART‐experienced pregnant PLHIV may have been misclassified as new ART starts if their prior ART history was outside of the Western Cape or if they started ART in years prior to the strengthened digitization of health records. PHDC pregnancy ascertainment has also improved substantially since 2013. Earlier pregnancies may be missing and pregnancies outside of the Western Cape are not captured [51], thus our indicator of the first pregnancy on record is likely an underestimate of true parity. In addition, we included only recorded live births in this study to maximize confidence in the pregnancy record and, therefore, cannot speak to issues of engagement in care in the context of pregnancy losses. We did not have reliable data on the date of HIV diagnosis, and data were not available regarding infant outcomes or infant feeding method which may have influenced postpartum outcomes. In this analysis, no outcomes after the first postpartum disengagement were considered and individuals were counted as having disengaged regardless of whether they subsequently returned to care. This was appropriate as even short interruptions in care during breastfeeding are a risk for HIV transmission. However, future research should examine patterns of engagement over longer periods.

## 5 CONCLUSIONS

There is growing recognition that even with increased access to lifelong ART, people are likely to cycle into and out of HIV care over time [52]. Whereas pregnancy used to be a vital point of first entry into HIV services, for many, it is now an important point of *re‐entry* into HIV care after a period of disengagement. Antenatal care coverage is extremely high in South Africa and many other high‐HIV burden settings, and this period presents an important opportunity for health interventions [53, 54]. Increased contact with routine health services during antenatal care should be leveraged to re‐engage pregnant PLHIV, understand prior ART experiences and barriers to care, and facilitate interventions to optimize sustained engagement in HIV care postpartum.

## COMPETING INTERESTS

KA and MAD received funding from ViiV Healthcare for an unrelated project. The other authors declare that they have no competing interests.

## AUTHORS’ CONTRIBUTIONS

TKP, MC, RD and RK conceptualized the analysis. NM contributed to data management. TKP analysed the data and drafted the manuscript. RK, NM, KA, LJ, JE, HM, LM, BHC, AB, MAD, MC and RDW provided iterative critical review on the analysis, revised the manuscript and appraised several drafts before approving the final version submitted for publication.

## FUNDING

Research reported in this publication was supported by the U.S. National Institutes of Health (NIH)’s National Institute of Allergy and Infectious Diseases (NIAID), the Eunice Kennedy Shriver National Institute of Child Health and Human Development (NICHD), the National Cancer Institute, the National Institute of Mental Health (NIMH), the National Institute on Drug Abuse, the National Heart, Lung, and Blood Institute, the National Institute on Alcohol Abuse and Alcoholism, the National Institute of Diabetes and Digestive and Kidney Diseases and the Fogarty International Center (FIC) under Award Number U01AI069924. TKP was supported by a Collaborative Initiative for Pediatric HIV Education and Research grant from the International AIDS Society, the FIC and NIMH (K43TW011943), VECD Global Health Fellowship funded by the Office of AIDS Research and FIC (D43 TW009337), and through the Fogarty IeDEA Mentorship Program. AB, LM and MAD were further supported by NICHD (R01 HD080465), and AB by investments from the Bill and Melinda Gates Foundation (1164272, 1191327). Additional investigator support is provided by the NIAID (K24 AI120796).

## DISCLAIMER

The content is solely the responsibility of the authors and does not necessarily represent the official views of the NIH or other funding agencies.

## Supporting information


**Table S1**. Characteristics of individuals with and without a CD4 cell count near first antiretroviral therapy (ART) initiation (within 6 months prior to ART initiation and 2 weeks after) and those who could and could not be linked to vital status records in the National Population Register (NPR).
**Table S2**. Three sensitivity analyses showing adjusted Cox proportional‐hazards models^1^ for time to first disengagement postpartum: (i) Cox proportional hazards model included all available pregnancies (*n* = 7309); (ii) Cox proportional hazards model with disengagement defined as 180 days with no evidence of HIV care (*n* = 6680); and (iii) Cox‐proportional hazards model including all women with a CD4 at first antiretroviral therapy (ART) initiation (*n* = 6588).
**Table S3**. In the subset of individuals with prior antiretroviral therapy (ART) history (*n* = 2584), estimated hazard ratios (HR) and 95% confidence intervals (CI) predicting first disengagement from care after delivery for the first pregnancy in the period April 2013–March 2019, including adjustment for time on ART at delivery.
**Table S4**. Estimated hazard ratios (HR) and 95% confidence intervals (CI) predicting first disengagement from care after delivery for the first pregnancy in the period April 2013 to March 2019, excluding observations with less than 2 years of postpartum observation time. Results were presented overall and in the subset of individuals with prior antiretroviral therapy (ART) history.
**Table S5**. Estimated hazard ratios predicting first disengagement from care after delivery for the first pregnancy in the period April 2013–March 2019, including people whose first antiretroviral therapy (ART) initiation record was in the 270 days prior to conception (*n* = 630 recent ART starts, *N* = 7310). Results are shown as crude and adjusted hazard ratios (HR) and with 95% confidence intervals (CI).

## Data Availability

The data that support the findings of this study are available on request from the corresponding author. The data are not publicly available due to privacy or ethical restrictions.
