# Supplementary material for: ART history prior to conception: trends and association with postpartum disengagement from HIV care in Khayelitsha, South Africa (2013–2019): a retrospective cohort study
Source: J Int AIDS Soc. 2024 Apr 2;27(4):e26236. doi: 10.1002/jia2.26236 (PMC10988117; doi:10.1002/jia2.26236)
Supplement: Supplementary file 1 — Table S1. Characteristics of individuals with and without a CD4 cell count near first antiretroviral therapy (ART) initiation (within 6 months prior to ART initiation and 2 weeks after) and those who could and could not be linked to vital status records in the National Population Register (NPR). Table S2. Three sensitivity analyses showing adjusted Cox proportional‐hazards models1 for time to first disengagement postpartum: (i) Cox proportional hazards model included all available pregnancies (n = 7309); (ii) Cox proportional hazards model with disengagement defined as 180 days with no evidence of HIV care (n = 6680); and (iii) Cox‐proportional hazards model including all women with a CD4 at first antiretroviral therapy (ART) initiation (n = 6588). Table S3. In the subset of individuals with prior antiretroviral therapy (ART) history (n = 2584), estimated hazard ratios (HR) and 95% confidence intervals (CI) predicting first disengagement from care after delivery for the first pregnancy in the period April 2013–March 2019, including adjustment for time on ART at delivery. Table S4. Estimated hazard ratios (HR) and 95% confidence intervals (CI) predicting first disengagement from care after delivery for the first pregnancy in the period April 2013 to March 2019, excluding observations with less than 2 years of postpartum observation time. Results were presented overall and in the subset of individuals with prior antiretroviral therapy (ART) history. Table S5. Estimated hazard ratios predicting first disengagement from care after delivery for the first pregnancy in the period April 2013–March 2019, including people whose first antiretroviral therapy (ART) initiation record was in the 270 days prior to conception (n = 630 recent ART starts, N = 7310). Results are shown as crude and adjusted hazard ratios (HR) and with 95% confidence intervals (CI). [file JIA2-27-e26236-s001.docx]

Supplementary Table 1. Characteristics of individuals with and without a CD4 cell count near first antiretroviral therapy (ART) initiation (within 6 months prior to ART initiation and two weeks after) and those who could and could not be linked to vital status records in the National Population Register (NPR).

|  | **Total** | **Has a CD4** | **No CD4** | **Linked to NPR** | **Did not link to NPR** |
| --- | --- | --- | --- | --- | --- |
| Number of individuals | 6680 | 6016 | 664 | 3168 | 928 |
|  |  |  |  |  |  |
| Age at ART initiation | 26.5 (23.2-30.4) | 26.6 (23.3-30.6) | 26.3 (23.0-30.0) | 26.9 (23.6-30.8) | 25.6 (22.3-29.9) |
|  |  |  |  |  |  |
| Year of delivery |  |  |  |  |  |
| April 2013 – March 2014 | 871 (13) | 813 (14) | 58 (9) | 629 (13) | 242 (14) |
| April 2014 – March 2015 | 1166 (17) | 1050 (17) | 116 (17) | 862 (18) | 304 (17) |
| April 2015 – March 2016 | 1180 (18) | 1065 (18) | 115 (17) | 919 (19) | 261 (15) |
| April 2016 – March 2017 | 1193 (18) | 1080 (18) | 113 (17) | 885 (18) | 308 (17) |
| April 2017 – March 2018 | 1367 (20) | 1228 (20) | 139 (21) | 957 (20) | 410 (23) |
| April 2018 – March 2019 | 903 (14) | 780 (13) | 123 (19) | 642 (13) | 261 (15) |
|  |  |  |  |  |  |
| ART history prior to conception |  |  |  |  |  |
| A. On ART | 1912 (29) | 1751 (29) | 161 (24) | 1238 (25) | 674 (38) |
| B. Returned | 381 (6) | 309 (5) | 72 (11) | 276 (6) | 105 (6) |
| C. Restart | 291 (4) | 263 (4) | 28 (4) | 212 (4) | 79 (4) |
| D. New start | 4096 (61) | 3693 (61) | 403 (61) | 3168 (65) | 928 (52) |
|  |  |  |  |  |  |
| Time-to-event outcome for first pregnancy in the study period |  |  |  |  |  |
| Disengaged (>270 days) | 2072 (31) | 1841 (31) | 231(34) | 1600 (33) | 472 (26) |
| Died | 28 (0.4) | 25 (0.4) | 3 (0.4) | 24 (0.5) | 4 (0.2) |
| Censored | 4580 (69) | 4150 (69) | 430 (65) | 3270 (67) | 1310 (73) |
|  |  |  |  |  |  |
| Has CD4 cell count at ART start | 6016 (90) |  |  | 4387 (90) | 1629 (91) |
| Median CD4 cell count nearest ART start date | 325 (217-475) |  |  | 325 (217-475) | 323 (216-474) |
| CD4 cell count category |  |  |  |  |  |
| <200 | 1291 (21) |  |  | 933 (21) | 358 (22) |
| 200-350 | 2066 (34) |  |  | 1512 (34) | 554 (34) |
| 350-500 | 1347 (22) |  |  | 982 (22) | 265 (22) |
| >500 | 1312 (22) |  |  | 960 (22) | 352 (22) |

Supplementary Table 2. Three sensitivity analyses showing adjusted Cox proportional-hazards models^1^ for time to first disengagement postpartum: i) Cox proportional hazards model included all available pregnancies (n=7309), ii) Cox proportional hazards model with disengagement defined as 180 days with no evidence of HIV care (n=6680), and iii) Cox-proportional hazards model including all women with a CD4 at first antiretroviral therapy (ART) initiation (n=6588)

|  | **i) All pregnancies (n=7309)** | **ii) Disengagement defined as 180 days with no evidence of HIV care (n=6680)** | **iii) Complete case model with available CD4 at first ART initiation (n=6588)** |
| --- | --- | --- | --- |
|  | **aHR (CI)** | **aHR (CI)** | **aHR (CI)** |
| ART history prior to conception |  |  |  |
| A. On ART | Ref | Ref | Ref |
| B. Returned | 1.97 (1.62-2.39) | 1.95 (1.60-2.36) | 2.09 (1.70-2.59) |
| C. Restart | 3.46 (2.92-4.09) | 3.31 (2.75-3.97) | 3.77 (3.15-4.51) |
| D. New start | 2.31 (2.05-2.60) | 2.16 (1.93-2.41) | 2.38 (2.10-2.71) |
|  |  |  |  |
| Age at delivery (per five additional years of age) | 0.80 (0.76-0.83) | 0.96 (0.95-0.97) | 0.95 (0.95-0.96) |
|  |  |  |  |
| First recorded pregnancy |  |  |  |
| No | Ref | Ref | Ref |
| Yes | 0.78 (0.71-0.86) | 0.86 (0.78-0.95) | 0.77 (0.69-0.85) |
|  |  |  |  |
| Log_10_ CD4 at first ART initiation |  |  | 0.97 (0.83-1.12) |

aHR – adjusted hazard ratio, CI – 95% confidence interval

^1^ Model (i) included cluster-robust standard errors to account for intra-individual correlation. Models (ii) and (iii) included a single pregnancy per individual, the first pregnancy in the period April 2013 to March 2019. Year of delivery was treated as a stratifying variable allowing the baseline hazard to vary by year and therefore is not shown.

Supplementary Table 3. In the subset of individuals with prior antiretroviral therapy (ART) history (n=2584), estimated hazard ratios (HR) and 95% confidence intervals (CI) predicting first disengagement from care after delivery for the first pregnancy in the period April 2013 to March 2019, including adjustment for time on ART at delivery.

|  | **Crude HR (CI)** | **Adjusted^1^ HR (CI)** |
| --- | --- | --- |
| ART history at pregnancy |  |  |
| A. On ART | Ref | Ref |
| B. Returned | 2.16 (1.73-2.68) | 2.49 (1.98-3.14) |
| C. Restart | 3.76 (3.04-4.66) | 3.84 (3.06-4.81) |
|  |  |  |
| Age at delivery (per five additional years of age) | 0.80 (0.74-0.87) | 0.87 (0.79-0.96) |
|  |  |  |
| First recorded pregnancy^2^ |  |  |
| Not first pregnancy | Ref | Ref |
| First pregnancy | 0.74 (0.61-0.89) | 0.74 (0.61-0.90) |
|  |  |  |
| Time from first ART initiation to delivery |  |  |
| 1.5-2.5 years | Ref | Ref |
| 2.5-3.5 years | 1.00 (0.80-1.25) | 0.86 (0.68-1.08) |
| 3.5-4.5 years | 1.04 (0.81-1.33) | 0.80 (0.61-1.03) |
| >4.5 years | 0.85 (0.68-1.08) | 0.67 (0.51-0.87) |
|  |  |  |
| Year of delivery |  |  |
| April 2013 – March 2014 | Ref |  |
| April 2014 – March 2015 | 0.72 (0.47-1.09) |  |
| April 2015 – March 2016 | 0.92 (0.63-1.35) |  |
| April 2016 – March 2017 | 0.88 (0.61-1.26) |  |
| April 2017 – March 2018 | 0.90 (0.63-1.28) |  |
| April 2018 – March 2019 | 0.39 (0.26-0.60) |  |

^1^In the adjusted model, year of delivery is not shown as it was treated as a stratifying variable allowing the baseline hazard to vary by year.

^2^First digital evidence of pregnancy based on all pregnancies recorded in the Provincial Health Data Centre.

Supplementary Table 4. Estimated hazard ratios (HR) and 95% confidence intervals (CI) predicting first disengagement from care after delivery for the first pregnancy in the period April 2013 to March 2019, excluding observations with less than two years of postpartum observation time. Results presented overall and in the subset of individuals with prior antiretroviral therapy (ART) history.

|  | **All deliveries April 2013-March 2018** | **Deliveries April 2013-March 2018 among women with prior ART history** |
| --- | --- | --- |
|  | **Adjusted^1^ HR (CI) n=5359** | **Adjusted^1^ HR (CI) n=1856** |
| ART history at pregnancy |  |  |
| A. On ART | Ref | Ref |
| B. Returned | 2.21 (1.77-2.77) | 2.55 (1.99-3.25) |
| C. Restart | 3.50 (2.81-4.36) | 4.01 (3.16-5.08) |
| D. New start | 2.24 (1.96-2.56) |  |
|  |  |  |
| Age at delivery (per five additional years of age) | 0.80 (0.76-0.84) | 0.88 (0.79-0.97) |
|  |  |  |
| First recorded pregnancy^2^ |  |  |
| Not first pregnancy | Ref | Ref |
| First pregnancy | 0.81 (0.73-0.91) | 0.73 (0.60-0.90) |
|  |  |  |
| Time from first ART initiation to delivery |  |  |
| 1.5-2.5 years |  | Ref |
| 2.5-3.5 years |  | 0.86 (0.67-1.09) |
| 3.5-4.5 years |  | 0.80 (0.61-1.05) |
| >4.5 years |  | 0.65 (0.49-0.87) |

^1^In the adjusted models, year of delivery is not shown as it was treated as a stratifying variable allowing the baseline hazard to vary by year.

^2^First digital evidence of pregnancy based on all pregnancies recorded in the Provincial Health Data Centre.

Supplementary Table 5. Estimated hazard ratios predicting first disengagement from care after delivery for the first pregnancy in the period April 2013 to March 2019, including people whose first antiretroviral therapy (ART) initiation record was in the 270 days prior to conception (n=630 recent ART starts, N=7310). Results are shown as crude and adjusted hazard ratios (HR) and with 95% confidence intervals (CI).

|  | **Crude HR (CI)** | **Adjusted^1^ HR (CI)** |
| --- | --- | --- |
| ART history at pregnancy |  |  |
| A. On ART | Ref | Ref |
| B. Returned | 2.10 (1.70-2.60) | 2.11 (1.71-2.61) |
| C. Restart | 3.62 (2.94-4.45) | 3.33 (2.70-4.10) |
| D. New start | 2.72 (2.40-3.08) | 2.40 (2.12-2.73) |
| E. Recent ART start before conception | 2.15 (1.80-2.57) | 2.01 (1.68-2.40) |
|  |  |  |
| Age at delivery (per five additional years of age) | 0.75 (0.72-0.78) | 0.79 (0.76-0.83) |
|  |  |  |
| First recorded pregnancy^2^ |  |  |
| Not first pregnancy | Ref | Ref |
| First pregnancy | 0.91 (0.82-1.01) | 0.82 (0.73-0.91) |
|  |  |  |
| Year of delivery |  |  |
| April 2013 – March 2014 | Ref |  |
| April 2014 – March 2015 | 1.04 (0.88-1.22) |  |
| April 2015 – March 2016 | 1.16 (0.99-1.36) |  |
| April 2016 – March 2017 | 1.03 (0.88-1.20) |  |
| April 2017 – March 2018 | 1.07 (0.91-1.25) |  |
| April 2018 – March 2019 | 0.65 (0.54-0.79) |  |

^1^In the adjusted model, year of delivery is not shown as it was treated as a stratifying variable allowing the baseline hazard to vary by year.

^2^First digital evidence of pregnancy based on all pregnancies recorded in the Provincial Health Data Centre.
